# Two complete mitochondrial genomes of *Myloplus rubripinnis* and *Metynnis hypsauchen* (Characiforme: Serrasalmidae)

**DOI:** 10.1080/23802359.2019.1687345

**Published:** 2019-11-08

**Authors:** Xiaolian Liu, Jufeng Jiang, Chunyan Li, Xiaohui Bai, Lin Ma, Keming Liu

**Affiliations:** Tianjin Fisheries Research Institute, Tianjin, China

**Keywords:** *Myloplus rubripinnis*, *Metynnis hypsauchen*, Mitochondrial genome, Phylogenetic analysis

## Abstract

*Myloplus rubripinnis* and *Metynnis hypsauchen* are two compressed-bodied ornamental fishes of Serrasalmidae family. In this study, complete mitochondrial genome sequences of the two species were determined. The mitogenomes were 16662 bp and 16737 bp nucleotides in length, and both contained 13 protein-coding genes (PCGs), 22 transfer RNAs (tRNA), 2 ribosomal RNAs (rRNA) and a control region. The phylogenetic tree revealed that *Myloplus rubripinnis* was closely related to *Myleus sp.* and *Myleus cf. schomburgkii,* while *Metynnis hypsauchen* was related to *Pygocentrus nattereri*, and then the two clades clustered into one group. Present mitogenome sequences of *M. rubripinnis* and *M. hypsauchen* will provide molecular information to the evolution and ecology studies of the two species.

*Myloplus rubripinnis*, belonging to Serrasalmidae in Characiformes, was native to the Amazon and the Orinoco river basins in South America, While *Metynnis hypsauchen* originated in Amazon and Paraguay river basins, as well as rivers of the Guiana Shield. They were introduced to China and had been popular aquarium fishes recently due to the silver compressed-body and sharp hook at the abdominal fin. Previous studies mainly focused on the biology (Kodisinghe and Jayamanne 2015), parasitology (Azevedo et al. 2012) and partial mtDNA sequence (Thompson et al. 2014). Less research was relative to the whole mitochondrial DNA gene.

Specimens were obtained from Tianjin Chaohong Recreational Fisheries co. LTD (117°21.28′E, 39°31.56′N) and deposited in Aquaculture Technology Research Laboratory of Tianjin Fisheries Research Institute, China (DLC801, DLC802). The mitochondrial DNA was obtained by Sanger sequencing and assembled by software Sequencher5.1.

The total mitochondrial genomes of *M. rubripinnis* and *M. hypsauchen* were 16662 bp and 16737 bp, with the GenBank accession No. MH358336 and MH358334. They were both consisted of 13 PCGs, 22 tRNAs, 2 rRNAs and a control region. The overall base composition were 29.01% A, 23.70% T, 31.01% C and 16.28% G, with AT content of 52.71% in *M. rubripinnis* and 29.11% A, 23.83% T, 30.84% C and 16.22% G in *M. hypsauchen*, being analogous to other fish species (Catanese et al. 2010; He et al. 2011). All PCGs started with an ATG codon except for COX1 (with a GTG start codon). Three types of complete stop codons were detected in open reading frames of *M. rubripinnis*: TAA for ND1, ATP8, ND4L, ND5, AGG for COX1, TAG for ND6, respectively. The remaining PCGs used incomplete codons (TA– or T–) as stop codons, which were completed via post-transcriptional polyadenylation that is common to vertebrate mitochondrial PCGs (Cheng et al. 2012; Niu et al. 2018). The codon usage characteristic in *M. hypsauche*n were similar with *M. rubripinnis*.

The phylogenetic position of the two species among Serrasalmidae was investigated using 18 mitogenomes by Bayesian and maximum likelihood. In our phylogenetic tree (Figure 1), *Myleus cf. schomburgkii* first clustered with *Myleus sp.*, then this clade clustered with *M. rubripinnis*. This group (*Myleus cf. Schomburgkii*, *Myleus sp.*, *M. rubripinnis*) was sister to the clade “true piranhas” comprising of *Pygocentrus nattereri* and *M. hypsauchen* with strong support.

**Figure 1.**
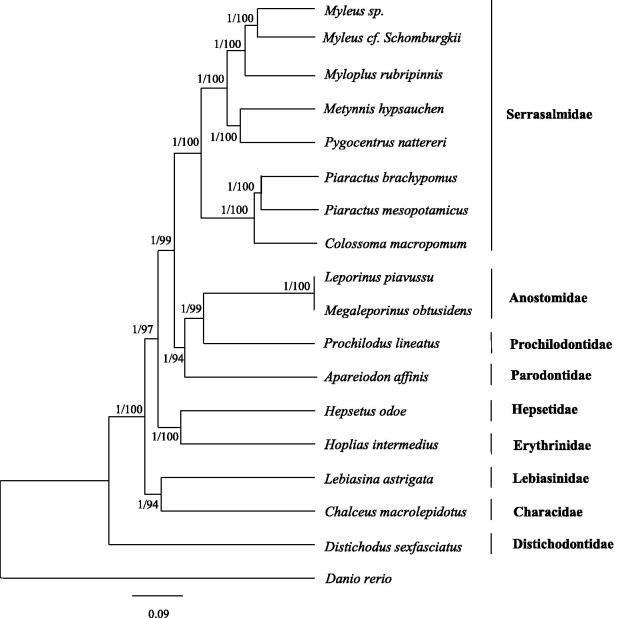
Phylogenetic tree among 18 species based on Bayesian and Maximum likelihood (ML) analysis. Numbers at nodes represent the posterior probability for Bayesian analysis and bootstrap value for maximum likelihood (ML) analysis. Genbank accession numbers: Myleus sp.(AP011997), *Myleus cf. schomburgkii* (MH358335), *Myloplus rubripinnis* (MH358336), *Metynnis hypsauchen* (MH358334), *Pygocentrus nattereri* (AP012000), *Piaractus brachypomus* (KJ993871), *Piaractus mesopotamicus* (KM245046), *Colossoma macropomum* (KP188830), *Leporinus piavussu* (KM886569), *Megaleporinus obtusidens* (KY825191), *Prochilodus lineatus* (KM245045), *Apareiodon affinis* (AP011998), *Hepsetus odoe* (AP011991), *Hoplias intermedius* (KU523584), *Lebiasina astrigata* (MH921292), *Chalceus macrolepidotus* (AB054130), *Distichodus sexfasciatus* (AB070242), *Danio rerio* (KM244705).
